# Comparison of renal effects of ibuprofen versus indomethacin during treatment of patent ductus arteriosus in contiguous historical cohorts

**DOI:** 10.1186/1472-6904-11-8

**Published:** 2011-06-30

**Authors:** Alla Kushnir, Joaquim MB Pinheiro

**Affiliations:** 1 Division of Neonatology, Department of Pediatrics; Albany Medical Center 43 New Scotland Avenue, Albany, NY 12208 USA; 2 Division of Neonatology, Department of Pediatrics; Cooper Hospital 1 Cooper Plaza, Camden, NJ 08103 USA

## Abstract

**Background:**

Ibuprofen treatment of patent ductus arteriosus (PDA) has been shown to be as effective as indomethacin in small randomized controlled trials, with possibly fewer adverse effects. However, adverse renal effects of ibuprofen have been noted in some trials and suspected in our practice.

The purpose of this study was to examine whether ibuprofen and indomethacin treatment of PDA have comparable effects on renal function as evidenced by urine output and serum creatinine.

**Methods:**

Retrospective chart review of 350 patients. Serum creatinine and urine output were recorded prior to start of treatment, during each course and after the last course of treatment. Pre-treatment mean creatinine and urine output values were compared to treatment and post treatment means using 2-factor repeated measures ANOVA.

**Results:**

165 patients were treated with indomethacin (2005-2006) and 185 received ibuprofen (2007-2008). There was no difference between treatment groups in demographics or baseline renal function. For both groups, the number of treatment courses was inversely correlated with birth weight and gestational age. Analysis of the first course including all patients, revealed significant increase in creatinine and decrease in urine output with both drugs, with a more pronounced effect of indomethacin on creatinine. In the subgroup of 219 patients who received only one treatment course, there was a significant increase in creatinine after indomethacin, but not after ibuprofen. In the 131 who received 2 or more courses, the decrease in urine output and increase in creatinine were not different between drugs. There were significant decreases in urine output observed in the second and third courses of ibuprofen treatment (both by 0.9 mL/kg/hr).

**Conclusion:**

Both drugs have a similar short-term effect on renal function. Indomethacin had a more prominent initial effect, while ibuprofen decreased renal function during the second and third courses similarly to indomethacin. The changes in renal function seen with ibuprofen treatment should be considered in fluid and electrolyte management, especially if treatment beyond one course is required.

## Background

Patent ductus arteriosus (PDA) is a common occurrence in very low birth weight (VLBW, ≤1500 g) infants, which often causes significant morbidities. Left-to-right shunting through the ductus may increase the risk of intraventricular hemorrhage [1,2], necrotizing enterocolitis [3], bronchopulmonary dysplasia, and death [4,5].

Successful pharmacological closure of PDA with indomethacin was first reported in 1976, with subsequent reports that indomethacin reduced neonatal morbidity [6,7]. However, indomethacin may lead to complications such as transient or permanent renal dysfunction [8,9], necrotizing enterocolitis, and reduced cerebral oxygenation [10]. These indomethacin-related complications have prompted researchers to seek safer pharmacological treatment for closure of PDA.

In recent years another cyclooxygenase inhibitor, ibuprofen, has been proposed for the treatment of PDA, and several randomized controlled trials have shown it to be as efficacious as indomethacin, with possibly fewer adverse effects [11]. It is thought that ibuprofen is better tolerated due to less effects on renal function, renal and mesenteric blood flow [12-14], and cerebral blood flow [15]. Adverse effects of ibuprofen have been noted in some trials [16] and suspected in our practice. This difference might be due to the fact that the infants in the previous trials were more mature (gestational age ~28 weeks) than the age of the infants at greatest risk for PDA (younger than 26 weeks); thus, it is difficult to extrapolate the clinical effects observed in those trials to the younger and unselected population typically treated in the clinical care context. Our primary objective was to ascertain whether ibuprofen and indomethacin treatment of PDA have comparable effects on renal function as evidenced by urine output and serum creatinine, during routine clinical usage.

## Methods

### Study design

This was a retrospective cohort study with a hypothesis that ibuprofen and indomethacin treatment of PDA have comparable effects on renal function as evidenced by urine output and serum creatinine. In October 2006 Neonatology staff switched from indomethacin to ibuprofen as the drug of choice for medical treatment of PDA. The cohort of neonates treated with indomethacin from January 2005 to October 2006 was compared to those treated with ibuprofen from October 2006 through December 2008. The study was approved by the Institutional Review Board of the Albany Medical Center, and exempted from requiring informed consent.

### Patient population

Records were reviewed for inborn or outborn preterm neonates born at any gestation and birth weight, admitted to the NICU of Albany Medical Center Hospital (New York State-designated Level IV Regional Perinatal Center) and requiring treatment for PDA, from January 2005 through December 2008. The clinical approach to the PDA was unchanged throughout the study period and it is summarized here. Prophylactic indomethacin was not used in our institution. Cardiology consultation was requested if a symptomatic PDA was suspected (extremely low birth weight neonate, widened pulse pressures, murmur, bounding pulses, or significant respiratory disease) and an echocardiogram was performed. The size of the PDA was described qualitatively by the cardiologists (as absent, small, mild, moderate, large, or synonymous terms), along with blood flow direction. Attending neonatologists generally initiated medical treatment if predominant left-to-right shunting was noted through a PDA larger than "small", while also considering the patient's clinical status, expected course given postnatal age, and potential contraindications. The day after the last treatment dose was administered, a follow-up echo was performed, and another course of either ibuprofen or indomethacin was given based on the same criteria used for the initial course. The decision to use further courses of medical treatment or resort to surgical ligation was made jointly by the attending neonatologist, cardiologist and cardio-thoracic surgeon. A mildly restrictive fluid therapy regimen was used, beginning with a weight-based guideline, and then tailored to each infant's condition and postnatal age. The fluid regimen was further restricted (by approximately 20 mL/kg/day below the individualized daily target) as cyclooxygenase inhibitor therapy was initiated.

Indomethacin was dosed at 0.2 mg/kg/dose once, then 0.1 mg/kg/dose IV daily for 2 more doses for neonates <750 g; 0.2 mg/kg/dose daily for 3 doses for neonates 750 g to 1 kg; and 0.2 mg/kg/dose every 12 hours for those over 1 kg. Ibuprofen was dosed at 10 mg/kg/dose once and then 5 mg/kg/dose daily for 2 more doses.

### Data collected

Demographic data were recorded into an Excel spreadsheet and de-identified. These data included gender, date of birth and date of discharge, birth weight, and gestational age. PDA-related data recorded included: date indomethacin or ibuprofen started, number of courses of medical treatment given, whether PDA was closed after the last treatment and whether surgical ligation of the PDA was performed.

Renal function prior to, during and after medical treatment of PDA was assessed by recording urine output and serum creatinine. Serum creatinine on the morning prior to administration of the first dose of ibuprofen or indomethacin was used as the baseline level. If no creatinine value was available from that day, the one from the prior day was used. During each treatment course, the last available serum creatinine was recorded. Resolution of the drug effect on renal function was assessed by evaluation of creatinine on the third to fifth day after the last drug dose.

Evaluation of urine output was done on the same schedule as creatinine, for 24 hours previous to, during treatment, and three to five days after the last dose of ibuprofen or indomethacin. Urine output was calculated as mL/kg/hour.

Secondary outcomes noted were necrotizing enterocolitis (NEC), bronchopulmonary dysplasia (BPD), and retinopathy of prematurity (ROP). NEC (defined using Bell's criteria [17]), spontaneous intestinal perforation (defined using Vermont Oxford Network criteria [18]), NEC-like illness, and whether NEC required surgical intervention were recorded. NEC-like illness was defined by the presence of clinical symptoms and radiographic appearance of bowel ischemia without pneumatosis or portal gas on abdominal radiograph. BPD was defined as oxygen requirement at 36 weeks post-menstrual age. ROP exam and the stage of retinopathy were also evaluated [19]. Presence of IVH and periventricular leukomalacia (PVL) were recorded, as well as the stage of IVH [20].

### Sample size and statistical analysis

An a-priori power analysis setting the α error at 0.05 and ß error at 0.20 revealed that a minimum of 50 patients in each group was required to detect a 20% difference in urine output - well within the expected size of the cohorts. SPSS for Windows (SPSS version 12.0.1, Chicago, IL), as well as Minitab (Minitab version 15.0, State College, PA) and Stata (Stata version 8, College Station, TX) were used to conduct statistical data analyses. Pre-treatment mean creatinine and urine output values were compared to treatment and post treatment means using 2-factor repeated measures ANOVA (generalized linear model, including terms for treatment, time, treatment-by-time interaction, and with patients nested within treatment group). Dunnett's post-hoc test was used to compare within-group changes in mean creatinine and urine output from baseline to each treatment course. A p value of < 0.05 was considered significant.

Chi-square analysis was performed to compare the proportion of patients experiencing the secondary outcomes of PDA closure, BPD, ROP, and NEC between treatment groups.

In order to assess whether there is a difference in the rate of PDA closure, number of doses of medical treatment required, or changes in renal function depending on gestational age or birth weight, these variables were evaluated in subgroup analyses. Gestational age categories were divided into: <25 weeks, 25 0/7-27 6/7 weeks, 28 0/7 - 29 6/7 weeks, and ≥ 30 0/7 weeks. Birth weight categories were divided into: ≤ 750 grams, 751 -1000 grams, 1001 - 1500 grams, and >1500 grams.

## Results

Of the 350 patients, 165 were treated with indomethacin and 185 received ibuprofen. The 7 cases where both drugs were used were excluded from the analyses of outcomes subsequent to the first course of treatment [Table 1].

**Table 1:** Baseline Population Characteristics

	Indomethacin	Ibuprofen
**Birth Weight, grams (mean)**	1048	1083
**Birth Weight Categories, n (%)**		
≤ 750 grams	54 (33)	56 (30)
751 - 1000 grams	38 (23)	41 (22)
1001 - 1500 grams	51 (31)	54 (29)
> 1500 grams	22 (13)	35 (19)
**Gestational Age, weeks (mean)**	27.7	27.8
**Gestational Age Categories, n (%)**		
< 25 weeks	33 (20)	34 (18)
25 - 27 6/7 weeks	61 (37)	68 (37)
28 - 29 6/7 weeks	35 (21)	37 (20)
≥ 30 weeks	36 (22)	47 (25)
**Gender**		
Male	82 (50)	108 (58) *
Female	83 (50)	78 (42)
**Number of courses, n (%)**	165	185
1	101 (61)	118 (63)
2	41 (25)	28 (15)
3	19 (12)	37 (20)
4	4 (2)	2 (1)
**Baseline urine output, mL/kg/h mean (SD)**	3.9 (1.4)	4.2 (1.6)
**Baseline creatinine, mg/dL mean (SD)**	0.96 (0.2)	0.93 (0.2)

PDA, patent ductus arteriosus

* denotes statistically significant difference between treatments, p < 0.05

The overall efficacy was the same for ibuprofen (71%) and indomethacin (68%) [Table 2]. The rate of ligation overall was the same after treatment with indomethacin and ibuprofen, with the exception of the neonates that required 2 courses of treatment for PDA closure. More babies required PDA ligation after receiving 2 courses of indomethacin (53.7%) compared to ibuprofen (28.6%) (p < 0.05) [Table 3].

**Table 2:** Secondary Outcomes According to Treatment Group

	Indomethacin	Ibuprofen	
	**(N = 161)**	**(N = 182)**	**p value**

**Day of Life at Start of Treatment (mean, SD)**	3.3 ± 2.3	3.5 ± 2.7	NS
**PDA Closed, n (%)**	109 (68)	129 (71)	0.4
**PDA Ligation, n (%)**	45 (28)	38 (21)	0.1
**BPD, n (%)**	72 (45)	90 (49)	0.2
**NEC-related conditions, any, n (%)**	27 (17)	32 (18)	0.8
NEC, n (%)	6 (4)	15 (8)	0.08
NEC-Like Illness (%)	13 (8)	5 (3)	0.03*
Spontaneous Intestinal Perforations, n (%)	8 (5)	12 (7)	0.5
**ROP, n (%)**	65 (52)	41 (30)	<0.001*
Severe ROP: Grades III-V	15 (12)	15 (11)	0.8
**IVH, any, n (%)**	58 (36)	57 (31)	0.4
Severe IVH: Grade III/IV	12 (8)	12 (9)	0.6
**PVL**	4 (2.4)	3 (1.6)	0.4
**Death, n (%)**	12 (7)	16 (9)	0.4

PDA, patent ductus arteriosus; BPD, bronchopulmonary dysplasia; NEC, necrotizing enterocolitis; ROP, retinopathy of prematurity; IVH, intraventricular hemorrhage; PVL, periventricular leukomalacia.

The denominator for the percent (%) for each secondary outcome reflects the number of patients with evaluation of that outcome - for ROP in particular, 82 infants did not have a retinal exam.

* denotes statistically significant difference between treatments, p < 0.05

**Table 3:** Treatment Efficacy by Number of Treatment Courses

	Indomethacin			Ibuprofen		
**Number of Courses Used**	**1**	**2**	**3**	**1**	**2**	**3**
**Total N**	101	41	19	118	28	37
**PDA Closed, n (%)**	89 (88.1)	18 (43.9)	3 (15.8)	108 (92.3)	15 (53.6)	7 (18.9)
**PDA Ligations**	8 (7.9)	22 (53.7)*	15 (78.9)	3 (2.6)	8 (28.6)*	26 (70.3)

PDA, patent ductus arteriosus.

Not all patients whose PDA remained open needed to have a surgical ligation.

* denotes statistically significant difference between treatments, p < 0.05.

Date of birth was considered as day of life one. There was no difference in the initial treatment day between indomethacin and ibuprofen [Table 4]. Second and third treatment courses started at a median of one day after conclusion of the previous course. There was, also, no statistical difference in the intervals between first and second (p = 0.2) or second and third treatment courses (p = 0.4) [Table 4]. There was no difference between drug treatment groups in gestational age, birth weight, or baseline creatinine or urine output. We found a significantly larger proportion of male infants in the ibuprofen group (58% males; p = 0.04) [Table 1]. However, ANOVA with gender as a covariate revealed no significant gender effect on baseline urine output or creatinine values, nor in their response to each treatment; cross-tabulations revealed no statistically significant differences between genders in the number of treatment courses, PDA closure or ligation rates in the ibuprofen group (data not shown). The number of treatment courses had a significant (p = 0.0001) inverse relation with both birth weight and gestational age, in chi-square analyses.

**Table 4:** Treatment Start Day

	Indomethacin	Ibuprofen
**Initial Day of Treatment**		
**Median**	3	3
**Mean**	3.3	3.5
**Minimum**	1	1
**Maximum**	18	22
**Interquartile range**	2	2
**Days between 1st and 2nd courses**		
**Median**	1	1
**Mean**	4.1	2.7
**Minimum**	1	1
**Maximum**	32	18
**Interquartile range**	2	0
**Days between 2nd and 3rd courses**		
**Median**	1	1
**Mean**	1.9	2.6
**Minimum**	1	1
**Maximum**	9	13
**Interquartile range**	0	2

**Figure 1:**
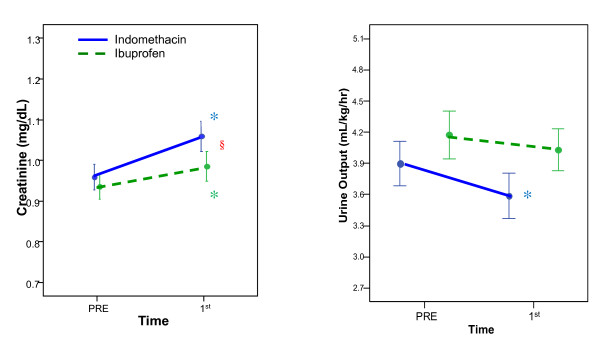
**Mean urine output and creatinine values for all patients by drug**. Comparison of mean urine output and creatinine values for all patients during the first course of therapy, by treatment drug. Indomethacin group (solid line) and Ibuprofen group (dashed line). PRE - pre-treatment baseline; 1st - end of first course of treatment. * denotes statistically significant (p < 0.05) change from treatment baseline; § denotes statistically significant (p < 0.05) difference in change from baseline between treatment groups.

When all patients were included in the analysis of the first treatment course, there was a statistically significant increase in creatinine (by 0.1 mg/dL) and decrease in urine output (by 0.3 mL/kg/hr) from baseline, after indomethacin [Figure 1][Table 5]. With ibuprofen treatment, only the increase in creatinine was significant. The ANOVA revealed that the increase in creatinine was significantly greater with indomethacin than with ibuprofen (p < 0.05 for both the treatment effect and the treatment-time interaction) [Table 5 and Figure 1]. However, there was no statistically significant difference in the effect of both drugs on urine output.

**Table 5:** Effect of Each Initial Treatment Course on Renal Function

	Indomethacin			Ibuprofen			Comparison between treatments on changes from baseline (ANOVA)	
	**Baseline**	**1st course**	**p value for change with indomethacin (t-test)**	**Baseline**	**1st course**	**p value for change with ibuprofen (t-test)**	**p value for main treatment effect**	**p value for treatment - time interaction**

Urine output (mL/kg/hr; mean ± SD)	3.9 ± 1.4	3.6 ± 1.4	0.033 *	4.2 ± 1.6	4.0 ± 1.4	*0.24*	*0.51*	*0.50*
Serum Creatinine (mg/dL; mean + SD)	0.96 ± 0.21	1.06 ± 0.24	<0.001*	0.93 ± 0.2	0.98 ± 0.24	0.005 *	0.039*	0.047 *

Supplemental analyses of the effect of each initial treatment course on renal function.

* denotes statistically significant (p < 0.05) change from baseline within a treatment group

**Figure 2:**
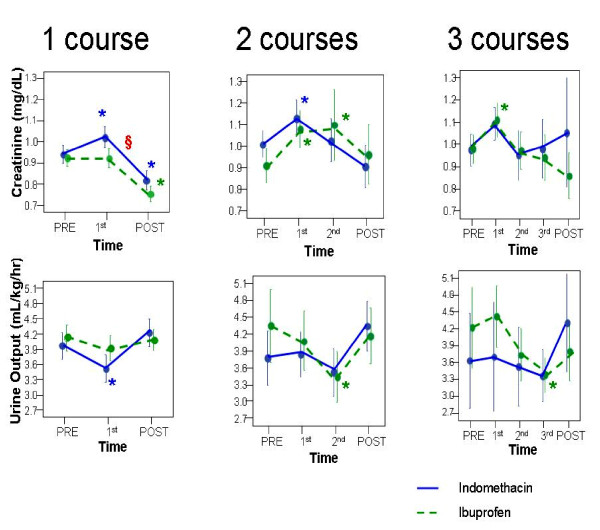
**Mean urine output and creatinine values by drug and number of courses**. Comparison of mean urine output and creatinine values by treatment drug for subgroups and defined by the total number of courses received. Left panel: patients who received only one course of either ibuprofen or indomethacin. Middle panel: patients who received total of two courses. Right panel: patients who received 3 total courses of medical treatment. Indomethacin group (solid line) and Ibuprofen group (dashed line). PRE - pre-treatment baseline; 1st - end of first course of treatment, POST - 3-5 days after the last course of treatment. * denotes statistically significant (p < 0.05) change from baseline within a treatment group. § denotes statistically significant (p < 0.05) difference in change from baseline between treatment groups.

In the subgroup of 219 patients who received only one course of therapy, there was a significant increase in creatinine (by 0.1 mg/dL, p = 0.001) after indomethacin, but not after ibuprofen [Figure 2], with a significant drug-by-time interaction (p = 0.029). Regarding urine output, ANOVA revealed no significant differences between drugs, although post-hoc tests showed a significant decrease in urine output only in the indomethacin group [Figure 2]. For the 131 neonates who received 2 or more total courses of treatment, there were significant decreases in urine output and increases in creatinine from baseline values, which were not significantly different between drugs [Figure 2]. Post hoc analyses revealed significant decreases in urine output from baseline observed during the last course of ibuprofen treatment (both by 0.9 mL/kg/hr, p < 0.05), in the subgroups receiving 2 or 3 courses [Figure 2]. Creatinine and urine output post-treatment returned to baseline values in both groups, regardless of the number of courses. Fluid intake was similar in both groups (data not shown).

There was no difference in secondary outcomes between the two drugs, except for ROP and NEC-like illness. There was a significantly higher rate of ROP in the indomethacin group [Table 2]. However, there was no difference in the rate of severe ROP between the groups. There was a significantly higher rate of NEC-like illness in the indomethacin group (8% vs. 3% for ibuprofen, p = 0.03); conversely, there was a trend towards more frequent diagnosis of NEC in the ibuprofen group (8% vs. 4% for indomethacin, p = 0.08) [Table 2], but no difference in the rate of spontaneous intestinal perforations between the two treatment groups [Table 2].

## Discussion

The purpose of this study was to examine whether ibuprofen and indomethacin treatment of PDA have comparable effects on renal function as typically used in a NICU, where it is common for ELBW neonates to receive more than one course of cyclooxygenase inhibitor therapy in order to close the PDA. In addition to the renal effects, our observations on less common potential adverse effects of cyclooxygenase inhibitors add substantially to the available information on safety of these therapies, as this is the largest comparison study conducted in the US examining efficacy and side effects.

Van Overmeire *et al. *studied the efficacy of indomethacin and ibuprofen given to larger premature infants (≤32 weeks) at the age of 2-4 days. They reported that the closure rate was similar (66% and 70%, respectively) after the first course and that there was no significant difference in side effects, although ibuprofen was associated with significantly less impairment of renal function [5]. The study showed that infants of lower gestational age (<28 weeks) had a lower pharmacological closure rate and underwent surgical ligation more frequently. This and several other studies [11,13,21-23] found that there was similar efficacy in PDA closure when using indomethacin or ibuprofen. Most of these reports found that ibuprofen had fewer side effects than indomethacin. However, these studies enrolled small numbers of neonates, that were more mature (mean gestational age >28 weeks) than those in our study. Van Overmeire described 40 preterm infants most of whom required a single course of medical treatment; only 7 required a repeat course [23]. Su *et al. *compared efficacy and side effects of ibuprofen and indomethacin in neonates (≤ 28 weeks) with echographically present PDA. They found both efficacy and side effect profile to be the same in both groups, except for a greater tendency toward oliguria after one course of indomethacin compared to ibuprofen [16]. Because surgical ligation was performed if the PDA was still present after one course, that study does not elucidate effects on renal function with further medical treatment.

The findings of our study confirm that indomethacin has a more prominent initial effect on measures of renal function than ibuprofen. However, ibuprofen had a detrimental effect on renal function in the second and third courses equal to that of indomethacin. Both drugs had a similar overall detrimental effect on renal function, with repeated courses.

Other recent studies support the notion that ibuprofen therapy is not devoid of renal effects in neonates [24-26]. Gournay *et al. *noted an increase in creatinine in the prophylactic ibuprofen group and in those who received a second course of ibuprofen, which resolved in the second week of life. They also noted a decrease in urine output with ibuprofen as compared with placebo that returned to baseline after the first course. Ticker and Yildirim described temporary oliguria and/or renal dysfunction after treatment with one course of ibuprofen that is similar to that seen with indomethacin. Vieux *et al. *found a significant decrease in glomerular filtration and tubular function impairment in the ibuprofen group that was not seen in the patients who did not receive ibuprofen [25]. Richards *et al. *reported that the effectiveness of ibuprofen in closing a PDA decreased with a second course, and that creatinine was significantly higher in neonates receiving a second course as compared to controls [27].

Our study also adds information pertaining to the safety of cyclooxygenase inhibitor treatment. There was no difference in morbidities potentially associated with cyclooxygenase inhibitor therapy between patients receiving ibuprofen or indomethacin, except for the incidence of ROP and NEC-like disease. These findings may be incidental to secondary analyses, or they may hint at clinically significant relationships. The higher rate of ROP in the indomethacin group was related primarily to higher incidence of stages 1 and 2 diseases, not of severe ROP. This has not been seen in previous studies [23,28,29]. It is possible that the difference in ROP rates is related to the more stringent oxygen saturation targets implemented in 2006 [30].

In the ibuprofen group there was a trend towards a higher frequency of NEC, but not of spontaneous intestinal perforations [Table 2]; NEC-like disease was significantly less frequent. There was no increase in the incidence of NEC seen in other studies of ibuprofen [5] and this finding is unexpected since ibuprofen does not significantly reduce mesenteric blood-flow velocity [13]. Diagnostic drift is unlikely since neither neonatology nor radiology staff physicians changed during the study period; furthermore, diagnostic criteria in the database remained constant. However, over the last decade there has been an increase in the incidence of NEC in a nationally representative sample of NICUs, and our observation may reflect this background trend [31].

Since this study compares two historical cohorts, it is possible that unmeasured confounders may have changed over time, and affected the primary or secondary study outcomes. However, in our NICU, indomethacin and ibuprofen were used for treatment of PDA during two immediately contiguous eras. Because the switch in management occurred through mutual agreement of all neonatologists, indomethacin was exclusively used before October 2006 and ibuprofen thereafter. In our analysis we excluded the few patients who received both indomethacin and ibuprofen. The study comprises only a 4 year time span, so the effects of therapeutic drift should be minimal; furthermore, there were no other planned or perceived changes in clinical management related to PDA.

The larger number of patients evaluated in this study increased our ability to detect important differences in adverse events between the two drugs. Most of the previous studies, both retrospective and prospective, had lower numbers of patients enrolled [5,11,14,16,23,29]. The retrospective nature of the study facilitated observation of the effects of both indomethacin and ibuprofen during unrestricted clinical usage. In particular, we demonstrated the consequences of repeated courses of cyclooxygenase inhibitor treatment in a large cohort of ELBW neonates.

This study confirms the findings or prior trials, revealing comparable effectiveness of indomethacin and ibuprofen in closing a PDA [11,13,21-23]. It also adds substantial weight to the notion that the likelihood of successful medical closure of the PDA diminishes with each subsequent course [27].

A limitation of our study is that it was retrospective. This allowed for some variability in treatment approaches by attending neonatologists; however, all clinicians used the same dosage regimen of PDA therapy and had similar approaches to PDA diagnosis. There was a wide range in the gestational age as well as acuity level of the neonates, which allows the results of this study to be more broadly generalizable. The larger proportion of males in the ibuprofen era is likely a chance finding, since review of our data on more than 1900 VLBW newborns over 14 years did not reveal a gender predilection of significant (treatable) PDAs or of PDA ligations. Furthermore, the gender difference between our study groups is unlikely to influence the results, since we found no gender-related differences in indices of renal function at baseline or in changes in urine output or creatinine during treatment courses.

## Conclusion

In summary, our data indicated that both indomethacin and ibuprofen appear to have a similar overall effect on renal function, particularly with repeated courses of therapy. This effect may be clinically apparent with decreased urine output and potential fluid overload, and it is more prominent during the first course of indomethacin. Whereas a decrease in urine output of nearly 10 mL/kg/day may be anticipated during the first course of indomethacin therapy for PDA, both indomethacin and ibuprofen will produce a decrease in urine output of about 20 mL/kg/day during subsequent courses. These changes in renal function should be considered and reflected in fluid and electrolyte management by prospectively decreasing fluid intake by approximately 20 mL/kg/day, especially if treatment is required beyond one course.

## Competing interests

Research support provided by an educational grant from Ovation Pharmaceuticals (Lundbeck, Inc.)

## Authors' contributions

JMBP participated in the design of the study, performed the statistical analysis, and helped draft the manuscript. AK conceived of the study, participated in its design and data acquisition, performed statistical analysis, and drafted the manuscript. All authors read and approved the final manuscript.

## List of abbreviations

PDA: patent ductus arteriosus; BPD: bronchopulmonary dysplasia; ROP: retinopathy of prematurity; NEC: necrotizing enterocolitis; IVH: intraventricular hemorrhage; PVL: periventricular leukomalacia; VLBW: very low birth weight

## Pre-publication history

The pre-publication history for this paper can be accessed here:

http://www.biomedcentral.com/1472-6904/11/8/prepub
